# Characterization of Mueller matrix elements for classifying human skin cancer utilizing random forest algorithm

**DOI:** 10.1117/1.JBO.26.7.075001

**Published:** 2021-07-05

**Authors:** Ngan Thanh Luu, Thanh-Hai Le, Quoc-Hung Phan, Thi-Thu-Hien Pham

**Affiliations:** a International University, School of Biomedical Engineering, Ho Chi Minh City, Vietnam; b Vietnam National University, Ho Chi Minh City, Vietnam; c Ho Chi Minh City University of Technology, Faculty of Mechanical Engineering, Ho Chi Minh City, Vietnam; d National United University, Mechanical Engineering Department, Miaoli, Taiwan

**Keywords:** human skin cancer, classification, Stokes–Mueller matrix formalism, random forest

## Abstract

**Significance:** The Mueller matrix decomposition method is widely used for the analysis of biological samples. However, its presumed sequential appearance of the basic optical effects (e.g., dichroism, retardance, and depolarization) limits its accuracy and application.

**Aim:** An approach is proposed for detecting and classifying human melanoma and non-melanoma skin cancer lesions based on the characteristics of the Mueller matrix elements and a random forest (RF) algorithm.

**Approach:** In the proposal technique, 669 data points corresponding to the 16 elements of the Mueller matrices obtained from 32 tissue samples with squamous cell carcinoma (SCC), basal cell carcinoma (BCC), melanoma, and normal features are input into an RF classifier as predictors.

**Results:** The results show that the proposed model yields an average precision of 93%. Furthermore, the classification results show that for biological tissues, the circular polarization properties (i.e., elements $m_{44}$, $m_{34}$, $m_{24}$, and $m_{14}$ of the Mueller matrix) dominate the linear polarization properties (i.e., elements $m_{13}$, $m_{31}$, $m_{22}$, and $m_{41}$ of the Mueller matrix) in determining the classification outcome of the trained classifier.

**Conclusions:** Overall, our study provides a simple, accurate, and cost-effective solution for developing a technique for classification and diagnosis of human skin cancer.

## 1 Introduction

According to the International Agency for Research on Cancer, there were 300,000 new cases of melanoma and over 1,000,000 new cases of non-melanoma skin cancer in 2018.^1^^,^^2^ Furthermore, the true number of skin cancer cases may be even higher than this figure due to many factors such as the registration methodology of skin cancer, the quality of skin cancer data.^3^ Without early detection and preventative control, melanoma can quickly develop and become far riskier, e.g., from stage I with a five-year survival rate of 97% to stage IV with a five-year survival rate of just 20% to 10%.^3^ Therefore, the early detection of skin cancer is essential in improving the prognosis of skin cancer patients.

No reliable biomarkers exist for melanoma diagnosis. Consequently, current diagnostic methods for skin lesions are subjective and imprecise. Typically, a patient must undergo around 36 biopsies to confirm (or discount) melanoma. However, despite this large number of biopsies, false negative predictions cannot be entirely ruled out.^4^ Thus, new skin cancer detection methods with greater accuracy and less invasiveness are urgently required. Among the various optical imaging technologies available nowadays, optical coherence tomography (OCT)^5^^,^^6^ and polarization-sensitive OCT^7^ make possible the real-time comprehensive morphological mapping of skin tissue samples with micrometer resolution by measuring the inherent properties of light (e.g., the scattering, birefringence, and refractive index properties) as it propagates through the sample.^8^ However, while OCT has a greater sensitivity for detecting melanoma than other techniques, such as reflectance confocal microscopy,^9^ high-frequency ultrasonography,^10^ and multispectral imaging,^11^ detecting early stage melanoma using OCT still poses a significant challenge^5^ due to the great number of different types of non-melanoma skin cancer.^12^

Many studies have shown that the Stokes–Mueller method, based on polarized light, has significant potential for replacing current clinical standards for skin cancer detection. Lu and Chipman^13^ proposed a Mueller matrix decomposition method for determining the diattenuation, retardance, and depolarization properties of a sample. Ghosh et al.^14^ investigated the efficacy of the Mueller matrix decomposition method in extracting the individual intrinsic polarimetry characteristics of a scattering medium with both linear birefringence (LB) and optical activity. Du et al.^15^ used a Mueller matrix imaging technique to construct two-dimensional images of the polarization parameters (i.e., attenuation, depolarization power, and linear retardance) of human skin basal cell carcinoma (BCC) and human papillary thyroid carcinoma tissues. Martin et al.^16^ used the Mueller matrix decomposition techniques proposed by Lu and Chipman^13^ and Ossikovski^17^ to differentiate between healthy and irradiated pig skin samples based on their measured retardance, diattenuation, and depolarization properties. Pham et al.^18^^–^^22^ employed a Stokes–Mueller method to examine the polarization properties of skin cancer, liver cancer tissues, neuroblastoma, collagen-rich tendons, and cartilage. It was shown that the proposed method yielded nine effective parameters for distinguishing between normal skin tissue and various skin cancer tissues, including BCC, squamous cell carcinoma (SCC), and malignant melanoma.

Machine learning provides a powerful tool for performing the objective and precise diagnosis of cancer through its use of statistics, probabilistic algorithms, and massive computational power. According to recent studies, machine learning techniques can improve 15% to 20% of the previous accuracy of cancer detection.^23^ For example, Codella et al.^24^ used a convolutional neural network (CNN) in deep learning combined with image segmentation algorithms to recognize melanoma in a dataset consisting of 900 training dermoscopic images and 379 test images. The classification accuracy was found to be 76%. By contrast, the average diagnosis accuracy of eight expert dermatologists was just 70.5%. Esteva et al.^25^ used a GoogleNet Inception v3 CNN architecture and a transfer learning technique to perform the first-level classification of three class disease partitions (benign, malignant, and non-neoplastic) with an accuracy of 72.1% and the second-level classification of the same partitions with an accuracy of 55.4%. Baldwin et al.^26^ proposed an automated Mueller matrix polarization imaging system and a classification and regression tree (CART) statistical analysis approach for classifying three classes of Sinclair swine tissue (normal, benign, and cancerous) and showed that the sensitivity was as high as 90%. Sigurdsson et al.^27^ detected five skin tumor lesion types using Raman spectra and a nonlinear neutral network. The experimental results showed that the proposed system achieved a classification rate of 80.5% for malignant melanomas and 95.8% for BCC. Legesse et al.^28^ used a perceptron algorithm to discriminate healthy and tumorous regions in BCC Stokes–Raman scattering (CARS) based on an analysis of the texture features. It was shown that the classifier achieved a sensitivity of 88% and a specificity of 91%. Murugan et al.^29^ used random forest (RF) and support vector machine (SVM) classifiers techniques for skin cancer detection. The experimental results showed that the proposed system achieved a classification rate of 72.2% using RF techniques and 87.81% using SVM+RF. Singh et al.^30^ detected breast cancer using RF classifier technique. It was shown that the classifier achieved a sensitivity of 90.56% and a specificity of 86.40%. Based on the fruitful achievement of Mueller matrix in Refs. 13–22 and machine learning techniques for skin cancer detection in Refs. 24–30, furthermore, the RF classifier is adopted for this study because of its advantage for overcoming the overfitting and suitable for classifying untrained data.^31^ Notably, the RF has the advantage in reducing the influence of noisy trees contribution.^32^ Moreover, the RF allows the ability of investigation to feature importance,^33^ which is useful to analyze the impact of optical properties of tissue on different types of skin pathology. Accordingly, the present study explores the feasibility for using a machine learning technique to discriminate between normal skin tissue and three classes of skin cancer based on the 16 elements of the Mueller matrix of a biomedical sample, in which all of the optical effects may appear simultaneously.

## 2 Stokes–Mueller Matrix Polarimetry Formalism for Skin Cancer Classification

The output Stokes vector, $S_{C}$, has the following form:

Sc=[S0S1S2S3]c=[Msample]S^c=[m11m12m13m14m21m22m23m24m31m32m33m34m41m42m43m44][S^0S^1S^2S^3]c, (1)

where [$M_{\text{sample}}$] is the Mueller matrix of a biomedical sample with depolarization, LB, circular birefringence (CB), linear dichroism (LD), and circular dichroism (CD) properties, and $S_{c}$ is the input Stokes vector.

Assume that the sample is illuminated by four input lights with linear polarization states (i.e., S^0°=[1,1,0,0]T, S^45°=[1,0,1,0]T, S^90°=[1,−1,0,0]T, and S^135°=[1,0,−1,0]T) and two input lights with circular polarization states (i.e., right-handed S^RHC=[1,0,0,1]T and left-handed S^LHC=[1,0,0,−1]T). The Mueller matrix elements of the biomedical sample, [$M_{\text{sample}}$], are then obtained as

$$[\begin{array}{cccc} m_{11} & m_{12} & m_{13} & m_{14}\\ m_{21} & m_{22} & m_{23} & m_{24}\\ m_{31} & m_{32} & m_{33} & m_{34}\\ m_{41} & m_{42} & m_{43} & m_{44} \end{array}]=\frac{1}{2}[\begin{array}{cccc} \left(S_{0\;\deg}(1)+S_{90\;\deg}(1)\right) & \left(S_{0\;\deg}(1)-S_{90\;\deg}(1)\right) & \left(S_{45\;\deg}(1)-S_{135\;\deg}(1)\right) & \left(S_{\mathrm{RHC}}(1)-S_{\mathrm{LHC}}(1)\right)\\ \left(S_{0\;\deg}(2)+S_{90\;\deg}(2)\right) & \left(S_{0\;\deg}(2)-S_{90\;\deg}(2)\right) & \left(S_{45\;\deg}(2)-S_{135\;\deg}(2)\right) & \left(S_{\mathrm{RHC}}(2)-S_{\mathrm{LHC}}(2)\right)\\ \left(S_{0\;\deg}(3)+S_{90\;\deg}(3)\right) & \left(S_{0\;\deg}(3)-S_{90\;\deg}(3)\right) & \left(S_{45\;\deg}(3)-S_{135\;\deg}(3)\right) & \left(S_{\mathrm{RHC}}(3)-S_{\mathrm{LHC}}(3)\right)\\ \left(S_{0\;\deg}(4)+S_{90\;\deg}(4)\right) & \left(S_{0\;\deg}(4)-S_{90\;\deg}(4)\right) & \left(S_{45\;\deg}(4)-S_{135\;\deg}(4)\right) & \left(S_{\mathrm{RHC}}(4)-S_{\mathrm{LHC}}(4)\right) \end{array}].$$ (2)

## 3 Skin Cancer Classification Model

### 3.1 Decision Tree Algorithm

Decision tree algorithms implement classification by splitting the dataset using binary questions based on the feature vectors.^34^ In particular, the feature vectors (denoted as X) are taken as tree nodes in the classification architecture, while the class labels are denoted as Y. A decision rule, d(t), is then used to map each X to d(X), where d(X) represents the class label of the feature vectors.^35^ Depending on whether or not the input features (i.e., attributes) satisfy the binary question, they are divided into two groups (known as branches) of nodes. Thus, by applying multiple questions to the flow, the decision tree classifies the input dataset into multiple different class labels.

One of the most well-known decision tree algorithms is the CART algorithm proposed by Breiman et al.,^36^ which constructs decision trees by applying a threshold for features that yield the best performance of the Gini index or information gain, respectively, depending on the tuned parameters.^37^ Notably, the algorithm not only accommodates both numerical and categorical variables but also handles outliers in the dataset in a robust manner.^38^ As such, it is ideally suited to the classification problem considered in the present study, in which the instances in the dataset [i.e., the Mueller matrix elements describing the optical (depolarization, LB, CB, LD, and CD) properties of human tissue samples] are numerical and have no missing values, but may contain outliers.

### 3.2 Random Forest Algorithm

The RF classification algorithm^31^ builds multiple individual sub-decision trees as building blocks for categorization tasks $\left\{T_{1}(X),T_{2}(X),T_{3}(X),\ldots T_{n}(X)\right\}$.^35^ Each individual sub-decision tree utilizes a different method to generate the binary questions used for classification purposes, and hence the resulting tree structure and organization are unique. Since each sub-decision tree in the RF architecture performs its own classification procedure, each tree can be regarded as an individual predictor and votes for the prediction of the input data and the final classification outcome can then be determined via a polling process. Compared to the traditional decision tree classification algorithm described above, the RF classifier provides a more effective reduction of the bias-variance by combining small decision trees with random feature subsets; thereby preventing overfitting during the training process.^39^

### 3.3 Gini Impurity

The Gini index is a statistical measure for quantifying the heterogeneity of a dataset.^40^ As described above, in binary decision trees, decision rules, d(t), are used to split the learning set of feature vectors L containing a certain number of feature vectors X. By splitting L into two sub-sets, namely $L_{1}$ and $L_{2}$, such that the data points of each subset conform to a specific rule, i.e., d(t). Consequently, the impurities of $L_{1}$ and $L_{2}$, respectively, are less than that of their parent, L.^41^ The impurity is measured by the Gini index, which has the following form:

$$G=1-\overset{k}{\underset{i=1}{\sum }}{\left(p(c_{i}|t)\right)}^{2},$$ (3)

where G is the Gini index at node t and p(t) is the probability of a given dataset L being assigned to class $c_{i}$. The Gini index varies from 0 to 1, where G=0 represents a complete equality of the data (i.e., all the data in the subset after splitting belong to a specific class label), whereas G=1 indicates a complete inequality of the data (i.e., none of the data in the subset after splitting belong to the same class label).

## 4 Experimental Setup and Data Acquisition Process

### 4.1 Experimental Setup

**Fig. 1:**
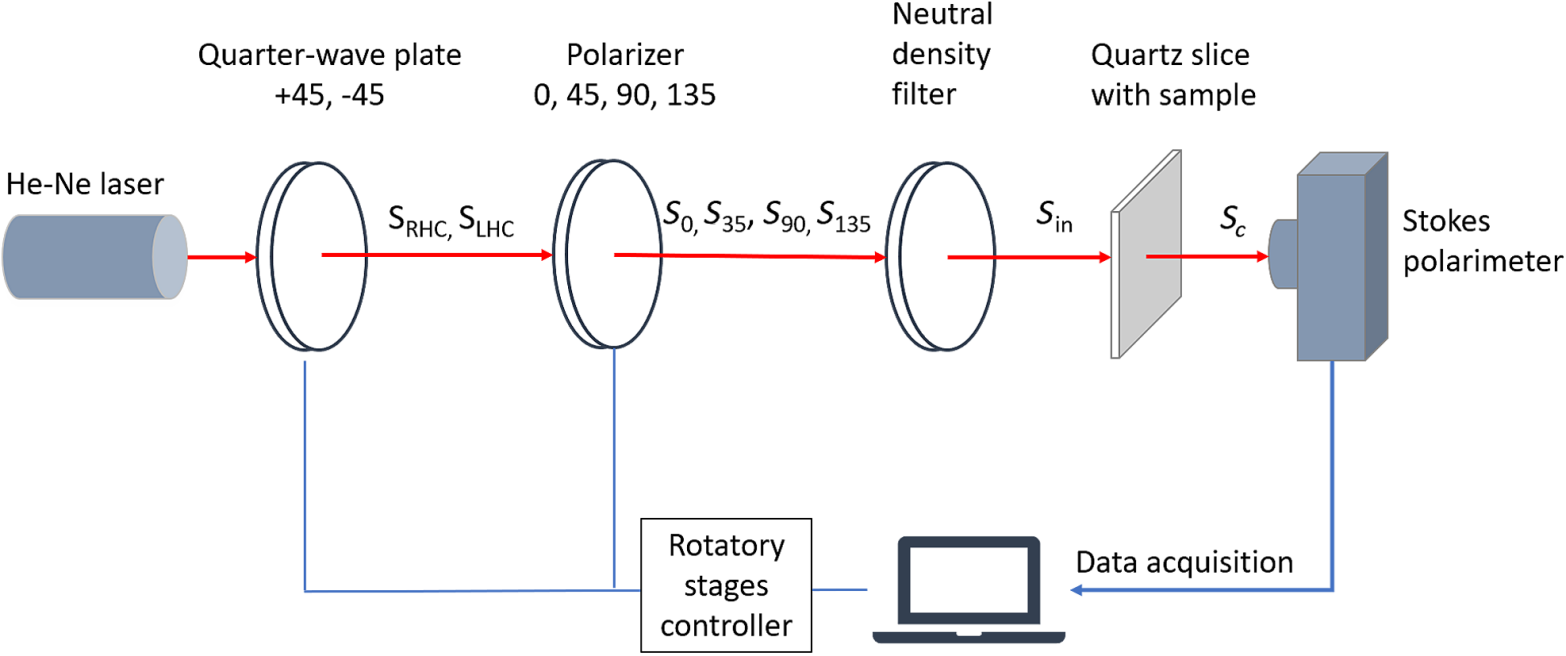
Setup of measurement system.

Figure 1 presents a schematic illustration of the experimental setup used in this study. The illumination light was produced by a frequency stable He-Ne laser (HNLS008R, SIOS Co.) with a center wavelength of 632.8 nm. The light emitted by the laser passed through a quarter-wave plate (QWP0-63304-4-R10, CVI Co.) and polarizer (GTH5M, Thorlabs Co.) and was then incident on the sample (i.e., biological tissue mounted on a quartz slice). It is noted that quartz slices were used to minimize the depolarization effect when light passed through the sample. Furthermore, the blank quartz slides were measured before performing experiments for calibration purposes. The quarter-wave plate was used to produce two circular polarization input states (right-handed and left-handed), while the polarizer was used to produce four linear polarization input states (0 deg, 45 deg, 90 deg, and 135 deg). For both optical elements, the polarization states were produced using rotary stages and a controller. The polarized light was passed through a neutral density filter (NDC-100-2, ONSET Co.) in order to ensure a consistent intensity of the light incident on the sample. The light emerging from the sample was detected by a commercial Stokes polarimeter (PAX5710, Thorlabs Co.), where the intensity was sampled at a rate of 33.33 (samples/s) and the output Stokes vector $S_{c}$ was constructed accordingly.

The experiments considered 32 biomedical skin tissue samples, specifically, 12 BCC samples, 4 melanoma samples, 4 normal samples, and 12 SCC samples, where each sample was cut into 4 to 6 slices. For each slice, the measurement process was performed at 4 to 6 different positions; with the output Mueller matrix calculated for each point. For each data point, the 16 elements of the Mueller matrix were compiled into a feature vector, X. Data from the 32 samples (i.e., 669 vectors) were split into two parts for training and testing. As shown in Table 1, the training dataset includes 607 feature vectors, where these vectors belonged to four different classes, namely BCC (282 vectors), SCC (231), melanoma (52 vectors), and normal (42 vectors). One sample of each skin tissue type was used for evaluation of the trained model; thus, the testing dataset includes 62 feature vectors, namely BCC (30 vectors), SCC (23), melanoma (3 vectors), and normal (6 vectors).

**Table 1:** Number of feature vectors of each class label in training and testing datasets.

	BCC	SCC	Melanoma	Normal
Number of training feature vectors	282	231	52	42
Number of testing feature vectors	30	23	3	6

It is noted that due to the difference in the shape of samples, each sample was under a different time of slicing and also the difference in measurement of interest position. Hence, the number of feature vectors belongs to each sample varies. This leads to the difference in the ratio of training and testing feature vectors for each type of skin tissue.

### 4.2 Classification Workflow

#### 4.2.1 Data preprocessing

One of the most common problems facing machine learning classifiers is that of imbalanced datasets, where the data records of the majority class overwhelm those of the other classes. In such a situation, the training process is unable to learn proper classification rules for the minority classes, and hence the classification accuracy for these classes is severely impaired.^42^ As shown in Table 1, the dataset employed in this study suffered this imbalance problem since the BCC class contained 282 feature vectors, whereas the normal class contained only 42 vectors. Accordingly, the oversampling technique^43^ was performed to randomly duplicate instances of the minority classes (SCC, melanoma, and normal skin) based on the original number of vector features belonging to the BCC majority class (see Table 2).

**Table 2:** Number of feature vectors of each class label in training and testing datasets after oversampling.

	BCC	SCC	Melanoma	Normal
Number of training feature vectors	282	282	282	282
Number of testing feature vectors	30	30	30	30

#### 4.2.2 Training

**Fig. 2:**
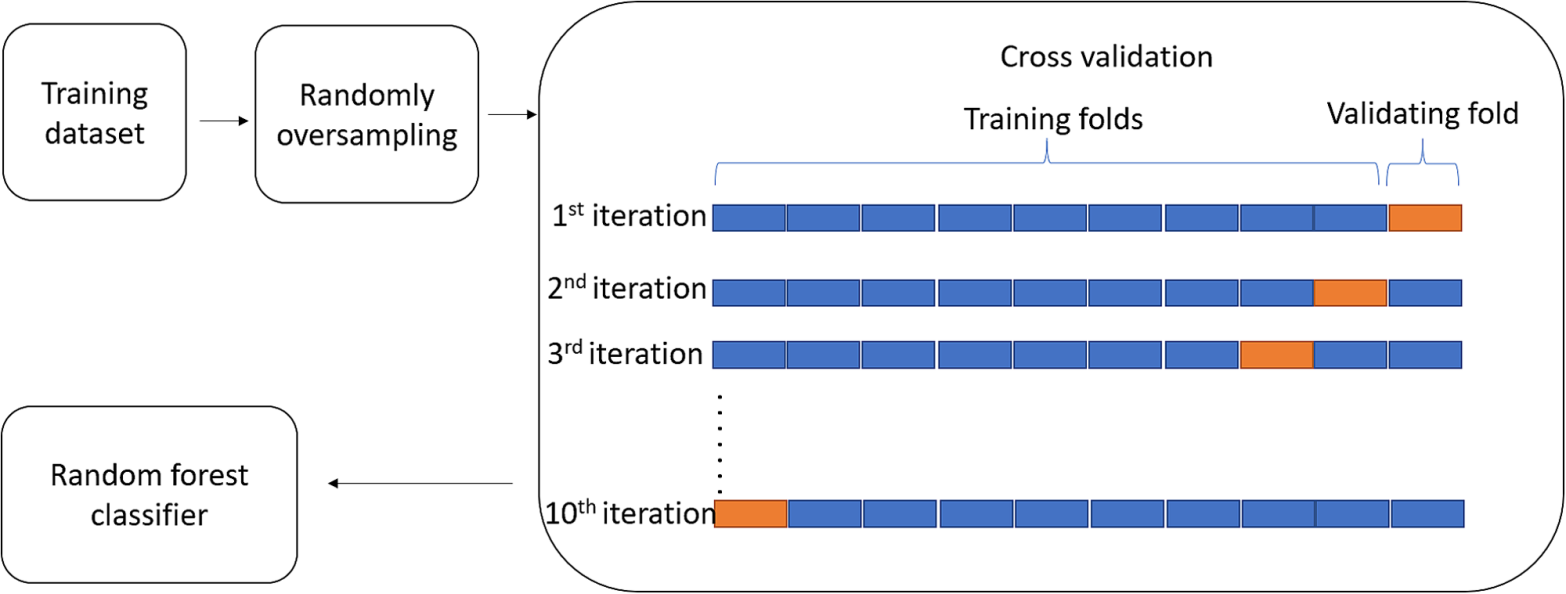
Flowchart showing RF classifier training process with 10-fold cross-validation.

Figure 2 presents a flowchart of the 10-fold cross-validation training process performed in this study. The selection of hyperparameters for the classification predictor was chosen with the help of the grid search technique. By feeding the ranges of hyperparameters into target predictors, the mentioned technique tries to fit each of the parameters into predictors to compute the score and select the best parameter for optimization of classification performance. Specifically, the range of number of trees and depth of each individual tree were [10, 20, 30…990, 1000] and [2, 3, 4… 20], respectively. The RF classifier consisted of 220 sub-decision trees, and the depth of the model was limited to 14 layers where these choices of parameters were made through experimentation. In addition, the classifier used the Gini impurity index as the splitting criterion. For each data point, 15 feature vectors were fed into the classifier. [Note that one of the features (Mueller matrix element $m_{11}$) was used for normalization purposes, and hence was not used as a predictor.) Notably, there were no instances of missing data, and thus handling schemes for missing data were not required. Cross-validation is usually performed using k=10 folds^44^ since a larger value of k reduces the size of each fold and thus reduces the difference in size of the training set and resampling subset, respectively. As a result, the bias, e.g., the difference between the true value and the expected value of the estimator, is decreased. For the present training process, 10-fold cross-validation was implemented with 3 times of repetition. According to Molinaro et al.^45^ and Kim,^46^ repeating k-fold cross-validation is beneficial in improving the precision score of classification models while maintaining a small bias.

## 5 Results and Discussion

**Fig. 3:**
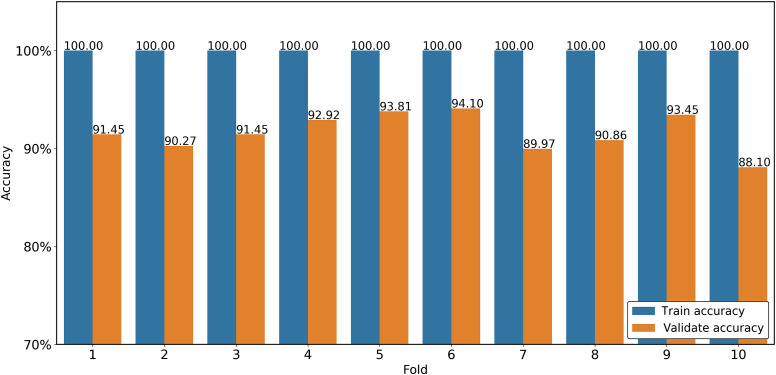
Training accuracy and validation accuracy of 10-fold cross-validation process.

Figure 3 shows the training and validation accuracy results for the 10 folds of the dataset. For the training set, the classification accuracy is equal to 100% in virtually every fold. By contrast, for the validation set, the classification accuracy reduces to around 91%. This tendency is reasonable since the oversampling process increases the number of duplicate data features, and therefore the trained model produces multiple rules for one instance, and the rules become specific for a portion of training data. This increases the training accuracy, but decreases the classification accuracy.

The performance of the trained RF classifier when applied to the test dataset, including 30 BCC, 23 SCC, 3 melanoma, and 6 normal feature vectors, then those of each class equals 30 after oversampling, was evaluated by a confusion matrix, as shown in Table 3. As shown, the optimal classification performance was obtained for the melanoma class, with 30 true positive cases, no false positive case or false negative case. A good classification performance was also obtained for the normal skin tissue, i.e., 30 true positive cases, no false negative cases, and just 2 false positive cases. However, for the BCC and SCC classes, the classification performance was degraded, with 7 false positive outcomes for the BCC class and 9 false negative outcomes for the SCC class. Interestingly, almost all, i.e., 7/9 of the SCC instances, were misclassified as BCC. It is noted that when cancerous tumors develop in the tissue, numerous changes in the collagen components occur, including the deposition of collagen fibrils resulting from an increased number of fibroblasts, the production of proteolytic enzymes for cancer invasion, etc.^47^^,^^48^ The change of biological structure that led the classification model significantly distinguished between normal tissue and cancerous tissue. Whereas some cases of BCC and SCC share the same clinical features, such as an ulcer with a rolled border, that may get the estimator confused.^49^

**Table 3:** Confusion matrix of trained RF classifier when applied to test dataset.

		Predicted class			
		BCC	Melanoma	Normal	SCC
True class	BCC	30	0	0	0
	Melanoma	0	30	0	0
	Normal	0	0	30	0
	SCC	7	0	2	21

The receiver operator characteristic curve is an evaluation metric for binary classification.^50^ It represents true positive rate (TPR) and false positive rate (FPR) at different thresholds. Thus, the calculation of the area under the curve (AUC) can be used to evaluate the model with unbiased estimation. The closer of the AUC score to 1, the better the model is. As shown in Table 4, the AUC score of melanoma and normal skin tissue was 1. The performance of the RF model on prediction BCC and SCC is lower; however, it is still a good score with 0.999 and 0.996, respectively. Overall, the mean AUC for all types of skin tissue is 0.999.

**Table 4:** AUC scores of each class and mean AUC of the proposed method.

BCC	SCC	Melanoma	Normal	Mean AUC
0.999	0.996	1.0	1.0	0.999

Table 5 analyzes the performance of the trained classifier for the four different class labels. The performance metrics, i.e., the precision, recall, and F1 score are defined as follows:

$$\text{Precision}=\frac{\mathrm{TP}}{\mathrm{TP}+\mathrm{FP}},$$ (4)

$$\text{Recall}=\frac{\mathrm{TP}}{\mathrm{TP}+\mathrm{FN}},$$ (5)

$$F1\text{score}=2\times \frac{\text{Precision}\times \text{Recall}}{\text{Precision}+\text{Recall}},$$ (6)

where TP is the true positive, FP is the false positive, and FN is the false negative.

**Table 5:** Performance evaluation analysis of trained classification model.

Class label	Precision	Recall	F1 score
BCC	0.81	1.00	0.90
Melanoma	1.00	1.00	1.00
Normal	0.94	1.00	0.97
SCC	1.00	0.70	0.82

The precision metric evaluates the prediction performance of the model, with a value closer to 1 indicating a better closeness of the predicted outcomes to the true outcomes. As shown, the classifier attains a precision of 1 for the SCC class. In other words, when supplied with the feature vectors of SCC, it correctly outputs a class label of normal in almost every case. Meanwhile, the recall metric evaluates the performance of the trained model for each individual prediction. In other words, the recall value of 1 for the BCC class indicates that if the trained model has previously predicted the current feature vectors as not belonging to the BCC class, then the current input belongs to the three other classes either SCC, normal, or melanoma with a probability of 100%. Finally, F1 score is the metric that combines precision and recall scores as harmonic mean. The F1 score takes both precision and recall scores into account, therefore, that is more general than these two metrics in evaluating models. Also shown in Table 4, the trained model achieves a good classification performance for the melanoma class (precision=1; recall=1). Moreover, the classifier also achieves a good performance for the normal class (precision=0.94; recall=1). However, as implied in the confusion matrix in Table 3, the classifier has a poorer performance for the BCC and SCC classes. Overall, the trained classifier successfully discriminates four classes of skin tissues with a (mean accuracy of 0.93).

**Fig. 4:**
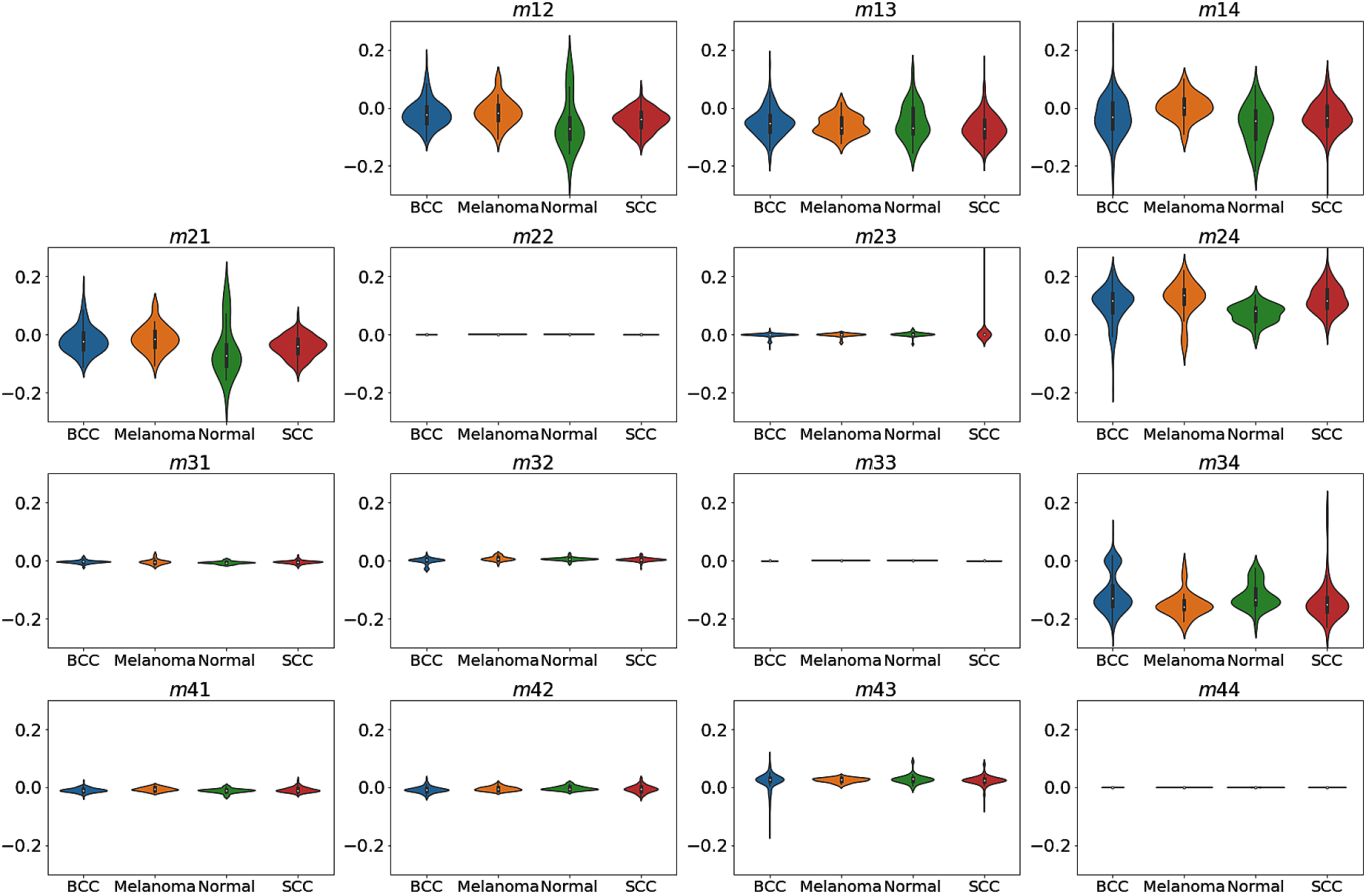
Magnitude and distribution of Mueller matrix elements for four different types of skin tissues.

**Fig. 5:**
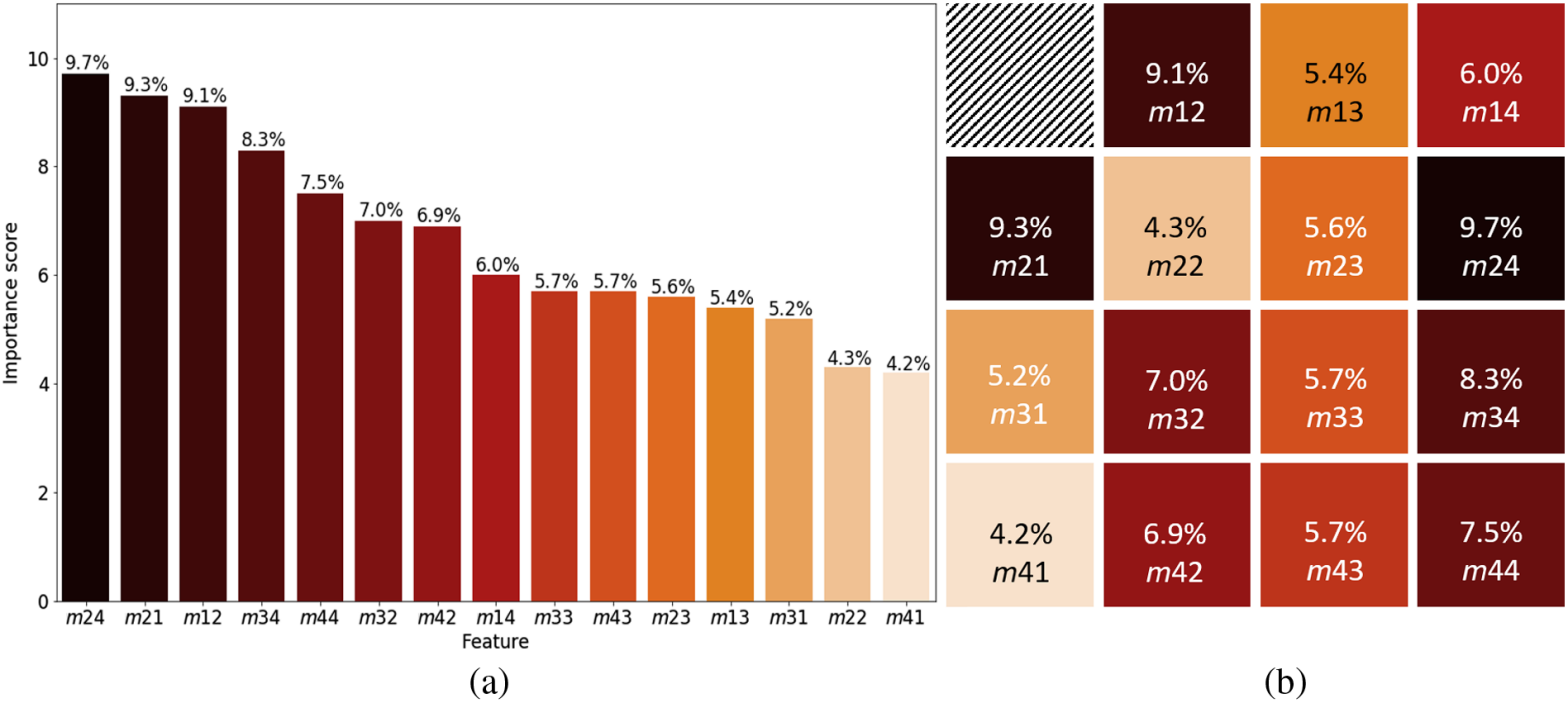
Feature importance ranking of 15 elements of Mueller matrix in (a) descending order; (b) Mueller matrix form.

Figure 4 presents the distributions and magnitudes of the 15 Mueller matrix elements of the SCC, BCC, melanoma, and normal skin tissue samples. The vertical and horizontal axes show the magnitude and distribution of the corresponding Mueller matrix elements, respectively. Note that, as described earlier, one of the matrix elements ($m_{11}$) was used for normalization purposes, and is hence omitted here. It is seen that for each Mueller matrix element, the magnitude is approximately equal for all four types of tissue sample. However, the distribution varies from one sample type to another. For example, for element $m_{22}$, the distributions of the different sample types are affected by outliers, which result in a significant skew of the distribution. Thus, element $m_{22}$ has only a low contribution to the outcome of the classification model, as shown in Fig. 5. In other words, although the range of data distribution varies among the four types of samples, it is difficult to classify the data without relying on advanced algorithms. It is also noted that the average standard deviation of the Mueller matrix elements $m_{12}$, $m_{13}$, $m_{14}$, $m_{21}$, $m_{24}$, and $m_{34}$ over 4 to 6 measurement points of the same slice are highest and deviated from 0.03 to 0.1 for four types of sampling. While the average standard deviation of the other elements over 4 to 6 measurement points of the same slice are smaller than 0.01. This is also clearly observed in Fig. 4 when the elements $m_{12}$, $m_{13}$, $m_{14}$, $m_{21}$, $m_{24}$, and $m_{34}$ have wider distributions than the other Mueller matrix elements.

Figure 5 shows the relative importance of the 15 different elements of the Mueller matrix within the classification model. Note that the feature importance represents the reduction in the node Gini impurity weighted by the node probability.^37^ For each decision tree, the importance score of feature i on node j, $ni_{j}$, is calculated as

$$ni_{j}=w_{j}G_{j}-w_{j}(L)G_{j}(L)-w_{j}(R)G_{j}(R),$$ (7)

where $w_{j}$ is the proportion of the number of samples reaching node j; $w_{j}(L)$ is the child node of the left split of node j; $w_{j}(R)$ is the child node of the right split of node j; and $G_{j}$, $G_{j}(L)$, $G_{j}(R)$ are the Gini impurities of node j and its left and right child nodes, respectively.

Thus, the importance of feature i in a specific tree, fi, can be calculated as

$$fi=\frac{\sum_{j}^{s}ni_{j}}{\sum_{k}^{N}ni_{k}},$$ (8)

where s is the number of node j splits for feature i; N is the number of nodes; and fi is the importance of feature i and is normalized to a value between 0 and 1.

Finally, the importance score of feature i in a forest of T estimators, Fi, is given by the average importance score of feature i over the individual trees, i.e.,

$$Fi=\frac{\sum^{T}fi}{T}.$$ (9)

As shown in Fig. 5(b) and referring to Eq. (2), elements $m_{24}$, $m_{34}$, $m_{44}$, and $m_{14}$ in the Mueller matrix, referring to the left- and right-handed circular polarization states, have high relative contributions of 9.7%, 8.3%, 7.5%, and 6.0%, respectively, toward the classification outcome. By contrast, elements $m_{43}$, $m_{33}$, $m_{23}$, and $m_{13}$, corresponding to the input linear polarization states of 45 deg and 135 deg, have relatively low contributions of 5.7%, 5.7%, 5.6%, and 5.4%, respectively. Similarly, elements $m_{32}$, $m_{42}$, $m_{31}$, $m_{22}$, and $m_{41}$, corresponding to linear polarization states of 0 deg and 90 deg also have low contributions of 7%, 6.9%, 5.2%, 4.3%, and 4.2%, respectively. Meanwhile, elements $m_{21}$ and $m_{12}$, corresponding to linear polarization states of 0 deg and 90 deg, have high relative contributions of 9.3% and 9.1%, respectively. In other words, the circular polarization elements in the Mueller matrix exert a greater effect on the classification outcome than the linear polarization elements. This finding is reasonable since skin tissue samples have a high natural scattering effect, which causes a helicity flip of the circular polarization light while passing through the sample.^51^ In general, the results presented above indicate that the proposed technique, based on Stokes–Mueller matrix polarimetry and an RF classification algorithm, provides a simple and well-accurate tool for skin cancer classification and diagnosis applications.

## 6 Conclusion

This study has proposed a Stokes–Mueller polarimetry method based on an RF classifier consisting of 220 sub-decision binary trees for discriminating between four different types of skin tissues, namely BCC, melanoma cancer, SCC, and normal, based on the measured values of the 16 elements in the output Mueller matrix. Based on the experimental results obtained for 32 skin tissue samples, it has been shown that the proposed model achieves an average classification accuracy of 93% for the four skin tissue types. It has additionally been shown that among all of the elements in the Mueller matrix, elements $m_{44}$, $m_{34}$, $m_{24}$, and $m_{14}$, relating to the left- and right-handed circular polarization states, respectively, have a stronger discriminatory power than those relating to the linear polarization states. Overall, the results show that the proposed framework has a promising potential for the development of machine learning approaches for automated cancer tissue screening and diagnosis.
